# A 3-D interactive microbiology laboratory via virtual reality for enhancing practical skills

**DOI:** 10.1038/s41598-024-63601-y

**Published:** 2024-06-04

**Authors:** Ebenezer Chitra, Siti Azreena Mubin, Vishna Devi Nadarajah, Wong Pei Se, Chew Fei Sow, Hui Meng Er, Nilesh Kumar Mitra, Vinesh Thiruchelvam, Fabian Davamani

**Affiliations:** 1 School of Health Sciences, International Medical University, Kuala Lumpur, Malaysia; 2 Asia Pacific University of Technology and Innovation, Kuala Lumpur, Malaysia; 3 School of Medicine, International Medical University, Kuala Lumpur, Malaysia; 4 School of Pharmacy, International Medical University, Kuala Lumpur, Malaysia

**Keywords:** Virtual reality, Virtual laboratory, Microbiology, Practical skills, Microbiology, Microbiology techniques

## Abstract

Virtual Reality (VR) laboratories are a new pedagogical approach to support psychomotor skills development in undergraduate programmes to achieve practical competency. VR laboratories are successfully used to carry out virtual experiments in science courses and for clinical skills training in professional courses. This paper describes the development and evaluation of a VR-based microbiology laboratory on Head-Mounted Display (HMD) for undergraduate students. Student and faculty perceptions and expectations were collected to incorporate into the laboratory design. An interactive 3-dimensional VR laboratory with a 360° view was developed simulating our physical laboratory setup. The laboratory environment was created using Unity with the (created) necessary assets and 3D models. The virtual laboratory was designed to replicate the physical laboratory environment as suggested by the students and faculty. In this VR laboratory, six microbiology experiments on Gram staining, bacterial streaking, bacterial motility, catalase test, oxidase test and biochemical tests were placed on the virtual platform. First-year biomedical science students were recruited to evaluate the VR laboratory. Students’ perception of the virtual laboratory was positive and encouraging. About 70% of the students expressed they felt safe using the VR laboratory and that it was engaging. They felt that the VR laboratory provided an immersive learning experience. They appreciated that they could repeat each experiment multiple times without worrying about mistakes or mishaps. They could personalise their learning by concentrating on the specific experiments. Our in-house VR-based microbiology laboratory was later extended to other health professions programmes teaching microbiology.

## Introduction

Immersive technologies such as virtual reality (VR) and augmented realitiy (AR) are becoming increasingly incorporated in school and university education, especially in the post-pandemic era to provide a pedagogical platform to enhance learning efficacy, accessibility and student engagement^1–3^. As educators increasingly recognize the potential of VR to enhance the pedagogical approaches, a variety of platforms have emerged, each contributing to the transformation of traditional classroom settings^4^. As we explore the multifaceted landscape of VR in education, we find that different platforms offer unique features tailored to specific educational objectives and subject matter catering to the distinct needs of learners across various educational levels. From immersive virtual classrooms to interactive simulations, these platforms help engage students in ways that extend beyond the limitations of traditional teaching methods^5^. Moreover, the adaptability of VR technology allows educators to craft specialised experiences that align with different subjects, fostering a more holistic and personalized learning environment^6^. This exploration will delve into a selection of educational VR platforms, highlighting their distinctive attributes and the pedagogical benefits they bring to the forefront. Whether through collaborative virtual spaces or hands-on scientific experiments, these platforms showcase the versatility of VR in addressing the diverse needs of learners and educators alike^7^. By examining these platforms and their applications, we aim to gain a deeper understanding of the transformative potential as well as challenges associated with the integration of VR into modern educational practices.

VR platforms allow the users to experience a realistic environment with visual, auditory and sometimes haptic stimuli. By manipulating the virtual objects to perform simulated tasks, the users get an immersive experience engaging multiple senses^8–10^. This keeps the students interested and engaged, making them active learners leading to enhancement of cognitive skills coupled with retention of information and improved performance^11–13^. Auditory cues play a pivotal role in creating a sense of presence and spatial awareness within virtual environments. The integration of 3D spatial audio not only immerses users in a realistic soundscape but also aids in conveying information and enhancing the overall learning experience^14^. Auditory stimuli can significantly impact memory retention and engagement in educational contexts, making it a valuable component in mulvvtisensory VR applications^15^. In the realm of education, VR transcends traditional pedagogical approaches by offering immersive, experiental learning. Virtual field trips, historical reenactments, and scientific simulations provide students with engaging and realistic educational experiences^16^. Studies indicate that VR-based learning experiences enhance knowledge retention and engagement, making it a valuable tool for educators^17^. One of the best examples of VR-based learning experiences in education is VR laboratories. Learning theories help us understand how students learn and help us to design effective learning experiences.

Learning theories can be used as a lens to understand the design and benefits of VR laboratories. Kolb’s experiential learning theory postulates that students learn by doing, which is the core pedagogy of VR-based laboratories, which are suitable for online as well as remote learning modalities^18^. Compared to traditional teaching methods, virtual applications gain more student satisfaction and self-efficacy as these engage the students in immersive and interactive learning experiences that might not be possible in the physical world^19–22^. Virtual laboratories are widely used in undergraduate education to allow students to safely practice the experiments in a virtual environment^23,24^. Skills training is crucial for undergraduate education since the students are exposed to the techniques for the first time and therefore require sufficient practice to achieve competency. VR laboratories are successfully used to carry out virtual experiments in basic sciences like organic chemistry^25^, biology^26^, chemistry^27^ and for clinical skills training in professional courses like medicine^8^, dentistry^28^, surgery^29^ and nursing^11^. These studies confirm that virtual simulators enhance skills acquisition as they enable the students to practice more and gain confidence as well as competence. Immersive technologies have facilitated the delivery of fully online bachelor’s degrees even in laboratory-intensive subjects like biology by providing virtual reality-based laboratory sessions^30–32^. The VR laboratories are safer as they reduce the risk of injury, biohazard and equipment damage^33^.

One of the main challenges in laboratory intensive programmes is the provision of sufficient practice time in the laboratory with formative feedback from the supervisor. Virtual laboratories are more beneficial for technical subjects like microbiology since they can reduce the risk of exposure to microbes, shorten experimental duration while providing active engagment with an immersive learning experience. They also allow the students to perform the experiments multiple times reducing the cost of infrastructure and consumables. Manipulating objects and performing virtual experiments enables the students to grasp the experimental principle and understand each step^18^. This was the basis for the design of our VR laboratory where the students can have practical experience through interactive simulations depicting the actual experiments. Studies designed and constructed based on learning theories produce engaging and interactive learning sessions that meet the intended learning outcomes^34^. We designed our virtual laboratory based on Kolb’s experiential learning theory and devloped it tailormade to our curriculum and actual laboratory protocols. The virtual microbiology laboratory was designed in-house by a multidisciplinary team with expertise in microbiology, education and computer science. This was introduced to undergraduate students undertaking the microbiology module in the biomedical science programme.

## Materials and methods

### Needs analysis

Focus group discussions (FGDs) were conducted with biomedical science students (n = 20) and with faculty (n = 5) teaching microbiology recruited by voluntary response sampling. For students, the inclusion criteria was biomedical science students in the first year and the exclusion criteria were (i) students from other programmes (ii) biomedical science students who were not in first year. For faculty, the inclusion criteria was faculty teaching microbiology in our university and the exclusion criteria was faculty not teaching microbiology in our university. FGDs were conducted guided by an interview guide. Open-ended questions were used to explore their expectations from a virtual reality laboratory and their concerns. The FGDs were transcribed *ad verbatim* and subjected to thematic analysis using the Braun and Clarke model to identify codes and form the final themes^35^. The main findings were incorporated into the design of the VR laboratory.

### Design and development of the VR laboratory

The VR laboratory was created using the Unity game engine providing immersiveness via VR head mounted display (HMD). Educators, subject matter specialists and developers collaborated to ensure an accurate representation of the laboratory environment and the experiments. The virtual laboratory was 3-dimensional and interactive providing a 360° view of the entire laboratory. The users would wear a headset with stereoscopic audio to provide audio-visual input and use hand-held controls for manipulating the virtual objects. The sensory stimuli correspond only to the virtual environment to give an immersive experience and promote experiential learning. The laboratory was designed to maintain fidelity mimicking the real environment, equipment and reagents. Real-life dimensions were maintained to simulate the actual physical laboratory. Realistic colours were used, and the experimental procedure was the same in both physical and virtual laboratories as per the feedback from the stakeholders.

### Implementation and evaluation

The VR laboratory with head mounted display was installed in the computers in the simulation centre and made available to the students through an online booking system. Undergraduate biomedical science students in the first year of their programme (n = 48) were recruited for the study by purposive sampling and informed consent was obtained. A knowledge questionnaire was designed, reviewed by the project team to ensure face and construct validity and further validated by a pilot study. This was administered to the students as a pre-test before the intervention and as a post-test after the intervention to evaluate the gain of basic knowledge relevant to the experiments.

Students’ perception of the VR laboratory experience was separately collected using a structured perception questionnaire with 5-point Likert scale. The perception questionnaire was adapted from a published study on virtual laboratories^36^. Focus group discussions were also conducted to get detailed feedback from the students on their VR experience. FGDs were transcribed *ad verbatim* and subjected to thematic analysis using the Braun and Clarke model following which codes were identified, grouped together, themes were generated and analysed^35^.

### Statistical analysis

Numerical data were analysed using SPSS. A P value of < 0.05 was considered statistically significant.

### Ethical approval

The project has been approved by the International Medical University Joint Committee on Research and Ethics (IMU 529/2021). All methods were performed in accordance with the relevant guidelines and regulations by the ethics committee.

## Results

### Educational design

**Figure 1:**
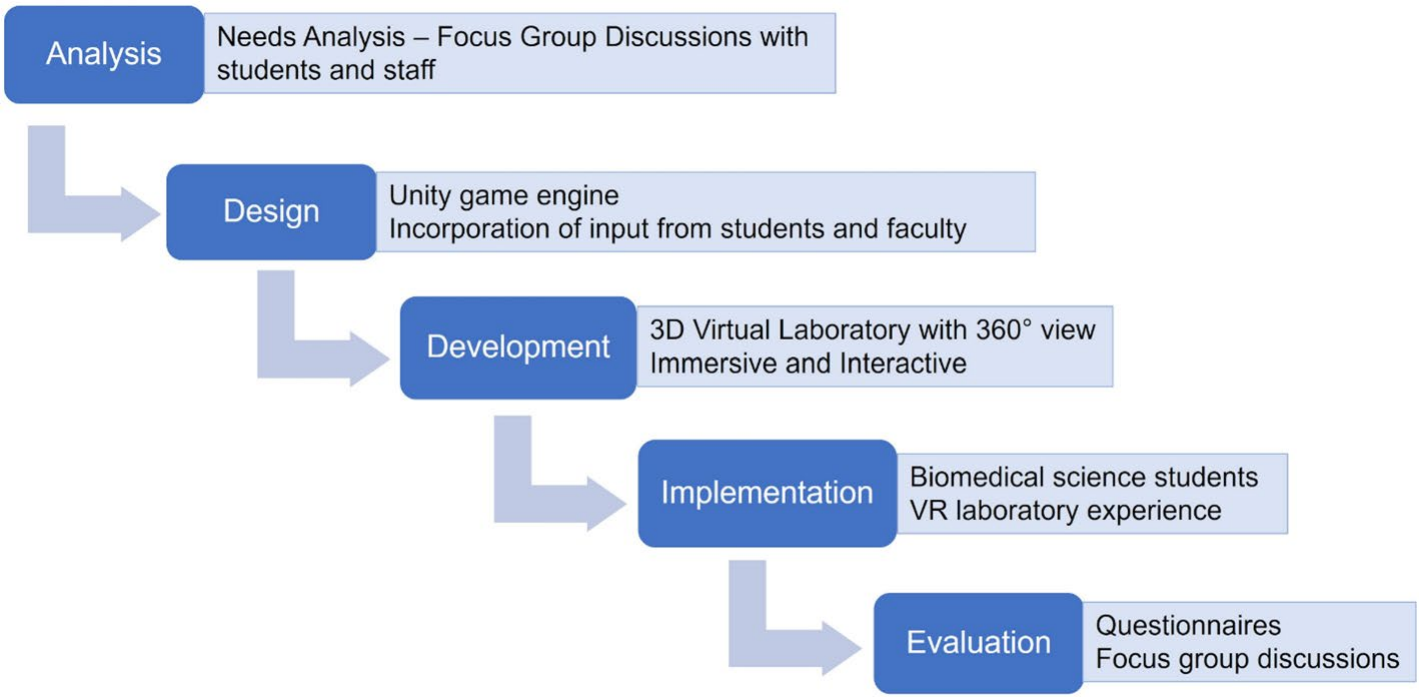
Phases of the VR laboratory project based on the ADDIE model Flow chart depicting the different phases of the VR laboratory project based on the ADDIE model: Analysis, Design, Development, Implementation and Evaluation.

The project was designed based on ADDIE model of instructional design involving analysis, design, development, implementation and evaluation^37^ (Fig. 1).

During the analysis phase, we conducted needs analysis to understand student and faculty perspectives and their expectations out of a VR laboratory. Their suggestions were incorporated into the design and development of the laboratory as described in the methods section. Upon launch of the VR laboratory, feedback and perceptions were collected the students through questionnaires and focus group discussions for evaluation.

### Needs analysis

From the focus group discussions conducted with students and staff, five major themes were identified viz., current practice, expectations from VR technology, suggested topics for VR, motivators for VR and barriers to using VR (Table 1). Students shared that their current practice to supplement practical classes was to watch YouTube videos featuring microbiology experiments. These videos, although they gave a general overview, featured different protocols not used in their actual practical classes. Some videos were quite old with varying quality and therefore not particularly useful. Both faculty and students welcomed the VR initiative.The students were excited about doing the experiments in the virtual world, which they felt would be safe without accidents or mistakes. They expected the VR laboratory to have up-to-date content that met the module learning outcomes. They expected visual appeal coupled with clear and concise narration of the procedure.

**Table 1:** Need analysis for the VR laboratory.

Themes	Feedback	Illustrative concept
Current practice	YouTube videos	Different videos depicted different protocols
		The quality of videos varied
Expectations from VR	Up to date	Use current protocol, Meet learning outcomes
	Clear narration	Explain the steps, Not too long
	Clear visual elements	
Suggested topics for VR	Laboratory skills	
	Morphology	Study morphology of microbes
	High risk specimens	Uncommon specimens, rare encounters
Motivators for VR	Infrastructure	Equipment
	Training	Trial runs
Barriers to use VR	Cost	Price of VR headset
	Technical capabilities	
	Health implications	Motion sickness, headache

Some barriers raised were related to the cost of VR headsets if students had to buy their own sets, the technical capability needed to develop and use VR and potential motion sickness or headache. Both faculty and students felt that the physical laboratory sessions should not be replaced, rather the VR laboratory should supplement the existing practical sessions. Nevertheless, both faculty and students agreed that VR laboratory development required testing and should be supported with adequate infrastructure and training. These findings were considered and incorporated into the design and development of the VR laboratory.

### Description of the VR laboratory

**Figure 2:**
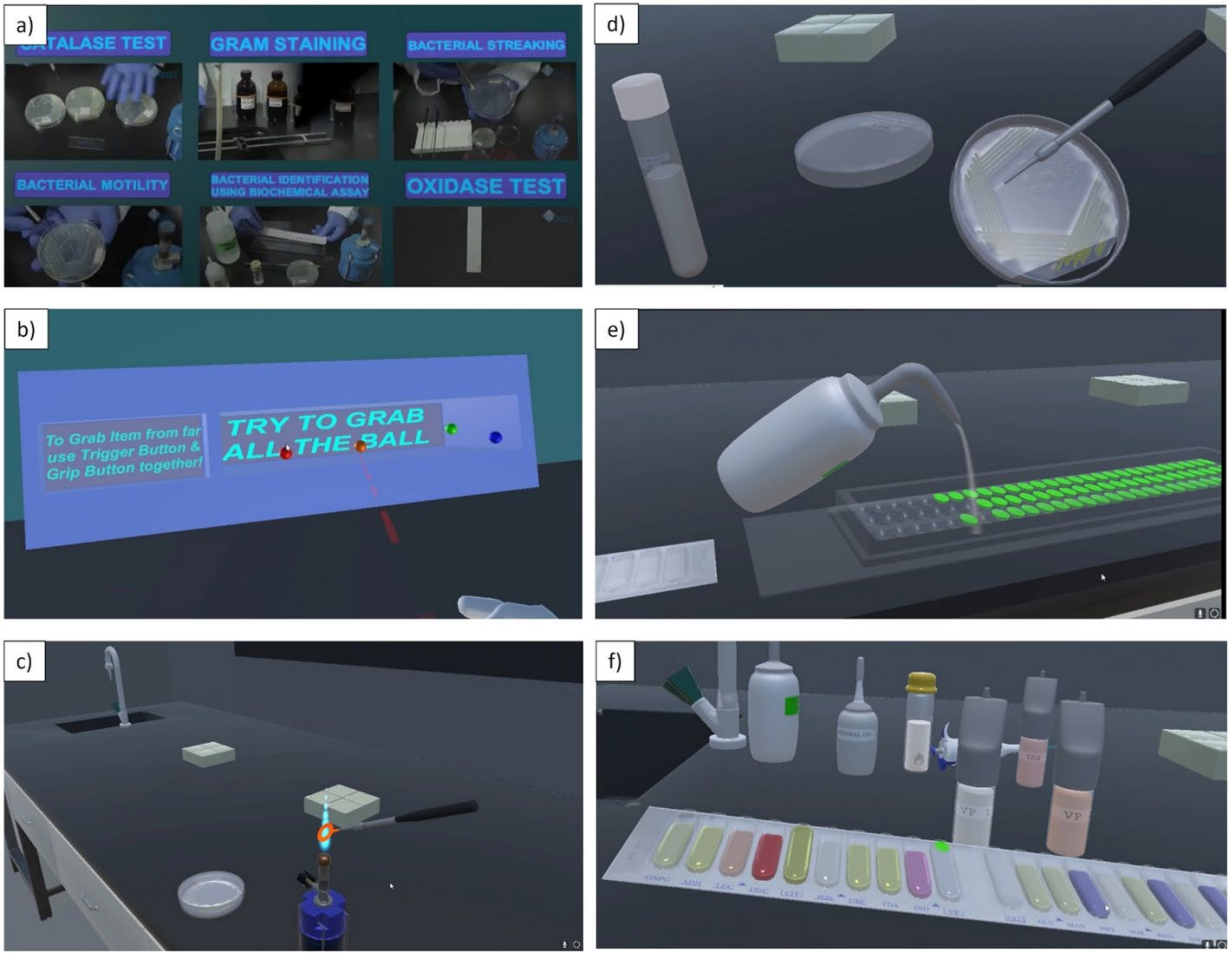
Screenshots of the VR Microbiology Laboratory. Screenshots depicting the VR microbiology laboratory. (**a**) The main lobby to choose the experiments, (**b**) A game on grabbing the different coloured balls to practice with the controls and (**c**–**f**) Snapshots of different experiments (**c**) Work table featuring the inoculation loop being sterilised (**d**) Picking bacterial colony from an agar plate (**e**) Filling water in the tray for API test (**f**) Biochemical identification of bacteria by API test.

The main lobby features a set of available experiments and an interactive game where the users can practice grabbing coloured balls to get used to the hand-held controls (Fig. 2a,b). There are six microbiology experiments on Gram staining, bacterial streaking, bacterial motility, biochemical assay, catalase test and oxidase test for the students to choose and practice (Fig. 2c–f). In the virtual experiemnts, the students could prepare a bacterial smear and use the reagents in the correct order to carry out Gram staining and see the result. They can pick bacteria with a loop and streak it on an agar plate. They can carry out the hanging drop method for visualizing bacterial motility under a microscope. They can do the biochemical test for bacterial identification using API strip and all the reagents with the results indicated by the colour change. The correct result appears on the wall. Catalase test is carried out on a slide with hydrogen peroxide solution to look for bubble formation. Oxidase strips are used and when the bacteria are placed on the strip, the colour changes for oxidase-positive bacteria.

When they chose an experiment, a brief introduction video played and then instructions appeared on the wall in front of them. The instructions are stepwise and they can proceed only after completing each step. The virtual laboratory is a replica of the physical laboratory they use in practical classes providing the same materials they use in the hands-on session. They could use tap water, Bunsen burner, microscope and all the standard materials in a microbiology laboratory. Gravity was featured with dropped objects falling down and liquids moving downwards. Prompts would appear on the equipment instructing them how to use them. Upon successful completion of the experiements, the results were displayed and a congratulatory message would appear. They could return back to the main lobby to choose another experiment (Supplemetary Information).

The protocols, materials and equipment used in our virtual laboratory are the same that are used in the physical laboratory for the experiments to ensure both are complementary. The VR laboratory was designed to complement the practical classes where the students could become familiar with the experiments, learn the protocols, understand the principles and reflect on their learning experience. Standard headsets were provided to all students to ensure that their VR experience was uniform.

### Student experience and feedback

#### Knowledge test

**Figure 3:**
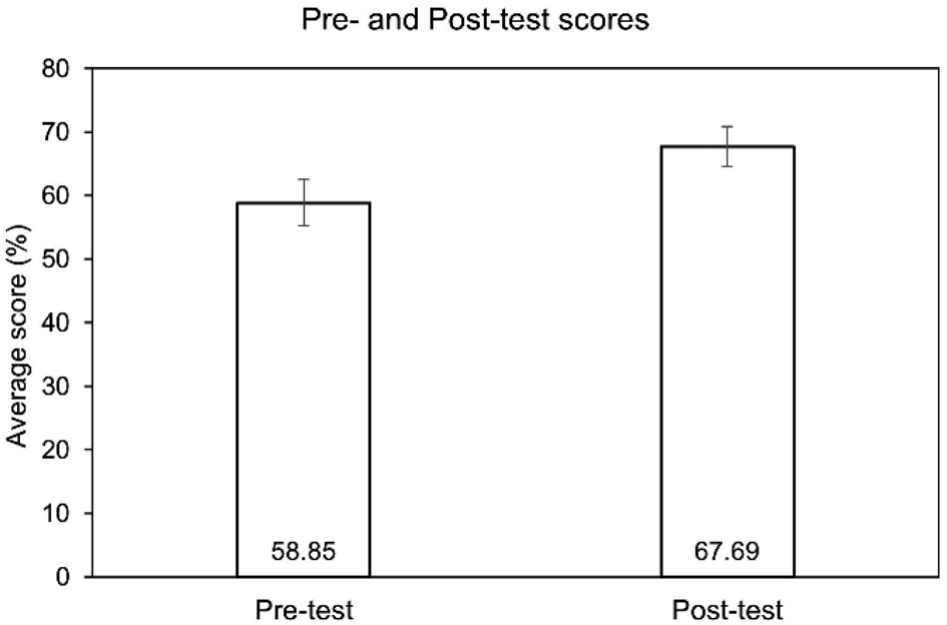
Pre- and Post-test scores of knowledge test. Graph depicting pre-test and post-test scores of knowledge test administered to the students (n = 26) who used the VR laboratory. Paired t-test gave a one-sided p value of 0.034.

To evaluate the pedagogical impact of the VR laboratory, pre-test and post-test on knowledge about the experiments were administered to the students before and after experiencing the VR laboratory. The mean test scores increased from the pre-test to the post-test even with a single VR experience. The mean pre-test score was 58.85 ± 1.86, and it increased to 67.69 ± 1.61 in the post-test (Fig. 3). Statistical analysis by paired t-test showed that the difference is statistically significant with a one-sided p value of 0.034.

#### Student perception of the VR laboratory

**Figure 4:**
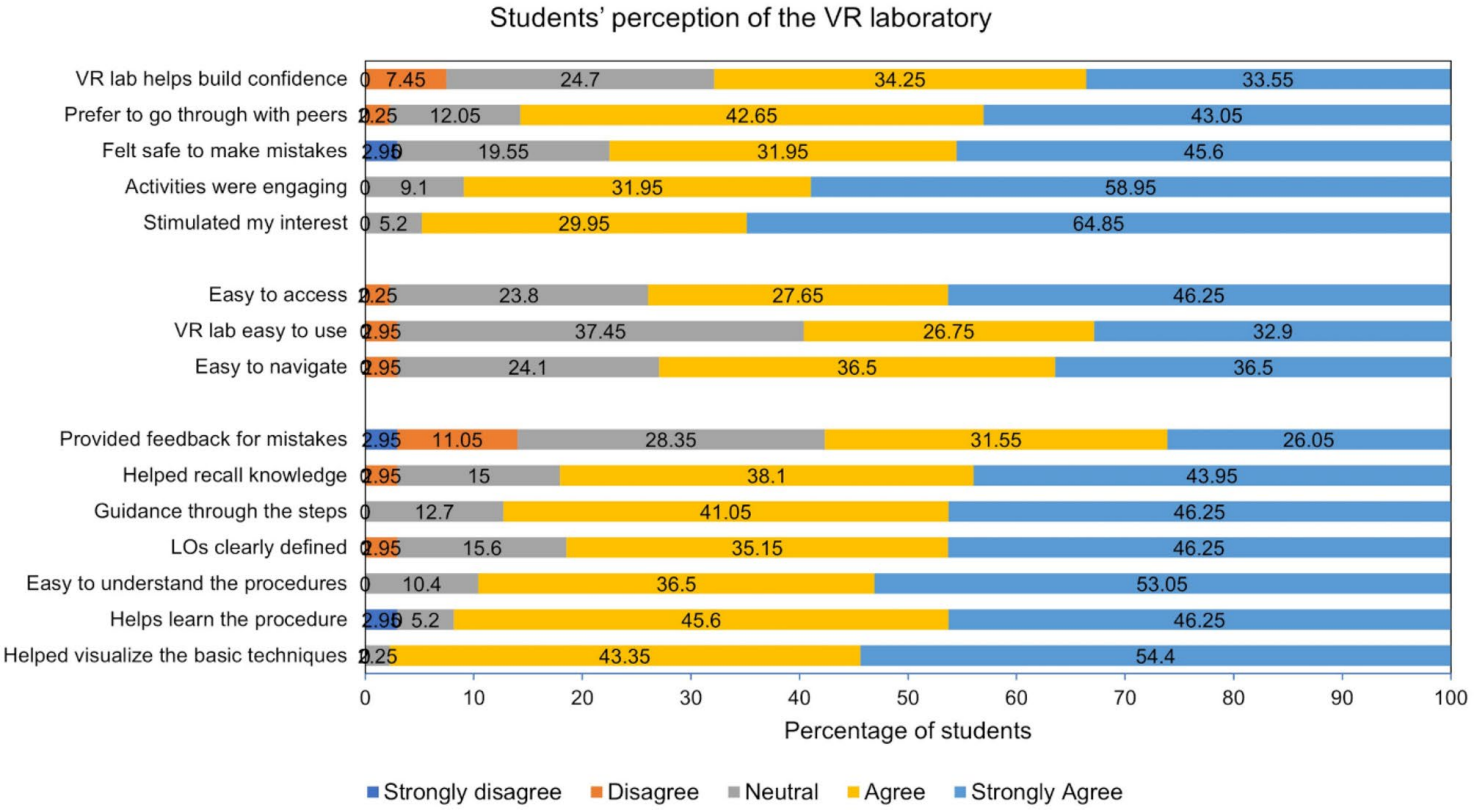
Student perception of the VR laboratory. Graph depicting student feedback collected on the ease of use, usefulness and experience with the VR laboratory.

We analysed how the students perceived this virtual laboratory using questionnaires and focus group discussions. Using a perception questionnaire, student feedback was collected on the ease of use, usefulness of the VR laboratory and student experience. Above 70% of the students agreed that the VR laboratory was easy to access and navigate and ~ 60% agreed it was easy to use (Fig. 4). The ease of use and navigation required getting used to the controls and therefore some students found it difficult. Access to the laboratory was provided through an online booking system which made it easy to access.

Most students (~ 90%) found it to be an engaging and interesting experience. They felt safe to make mistakes in the virtual environment and most students (~ 60%) agreed it helped build confidence. Most students (~ 90%) agreed that the VR laboratory was useful, helped them learn and understand the procedure, and recall knowledge as sufficient guidance was given. They were able to learn the techniques and visualize the procedure as well. A few students preferred to go alone to practice in the VR laboratory while the others preferred to work in a group.

#### Feedback through focus group discussion

In the focus group discussions, students’ feedback on their VR experience, features of the VR laboratory, advantages and limitations was collected and tabulated (Table 2). The students found the VR experience to be immersive and quite real like playing a game. They liked the guidance given through stepwise instructions and the synchronisation to duplicate the physical laboratory. The students especially appreciated that the they were able to make mistakes and repeat the experiments multiple times without wasting the reagents. When they made a mistake, they could correct it and only then proceed to the next step. This helps them when they enter the real laboratory to avoid such mistakes. Students were cautioned about motion sickenss and a couple of students who experienced motion sickness were immediately rested. Some students had difficulty manoeuvring the hand-held controls, which required some practice time. Overall, our VR microbiology laboratory was found to be effective and useful for building practical competencies in microbiology.

**Table 2:** Student feedback on their experience in using the VR laboratory.

Themes	Feedback	Illustrative concept
Experience	Fun, Cool, like playing a game	Headset gives a spectacular view
	Immersive, quite real	Feels like actually being in the laboratory
Features	Clear instructions; Helps reinforce the knowledge	Can understand the principle and get familiar with the procedure
	Accurate details synchronised to the real laboratory	
	Trial game is useful to get used to the controls	
Advantages	Time saving	Real experiments take a long time
	Can repeat multiple times	There is no wastage of reagents
	Can make mistakes	In the VR laboratory, if a mistake is made cannot proceed to the next step. Can correct the mistakes made in the VR laboratory while doing the real experiments
Limitations	Motion sickness, dizziness	
	Hand-held controls difficult to use	
	Difficult to use with spectacles	
	Haptic response not included	

## Discussion

**Figure 5:**
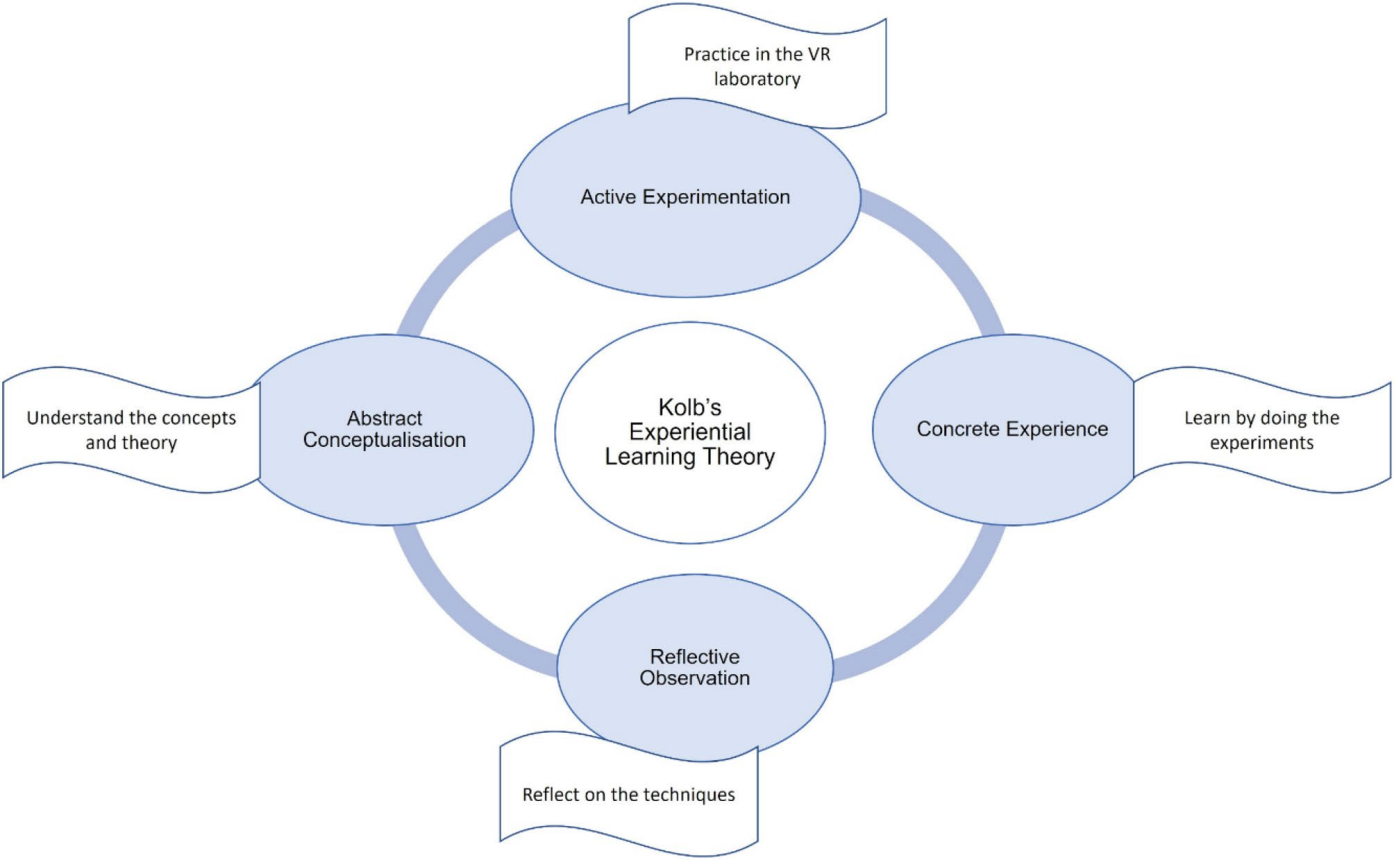
Kolb’s experiential learning theory. Diagram depicting the application of Kolb’s experiential learning theory to the students’ learning experience using the VR laboratory.

VR technology has emerged as a powerful tool that can help create these immersive and engaging learning experiences to students. VR applications are increasingly used to practise clinical and laboratory skills in medical, nursing, and basic science programmes. Most studies support the concept that virtual environments promote motor learning^20,38–40^. A study conducted in Denmark showed that using virtual laboratory simulation helped the learners connect theory with practice, learn the procedures and techniques while enhancing their engagement and interest^22^. Applying Kolb’s experiential learning theory to our VR laboratory experience, when the students actively experiment in the virtual laboratory, they build concrete learning experiences and as they reflect on their experiments and observations, they can understand the concepts and improve in the next cycle (Fig. 5). Virtual laboratories are even seen as supplement for traditional teaching activities. Our VR laboratory with head mounted display is designed to promote asynchronous learning of practical skills, which normally are delivered through face to face sessions. Scaffolding guidance is an instructional technique that involves providing stepwise instructions for carrying out the experiment at every step^41^. This was adopted in our laboratory design as it is known to enhance procedural knowledge and support the students in an adaptive manner as they can touch and manipulate the simulated objects that gives a realistic feel^42^. It takes the pressure off students to remember the protocol and enables them to fully focus on the experiment.

VR laboratories are useful especially for undergraduate learning where the focus is on getting familiar with the protocol and understanding the concepts. Using VR simulation for psychomotor skills training is shown to be highly beneficial in training students and teaching procedural knowledge. There is strong evidence to suggest that motor skills could be improved through virtual laboratories^43–46^. A study on virtual microbiology simulation reported that students who used the VR technology scored significantly higher than those who used traditional learning methods^8^. A virtual microbiology laboratory developed for pharmacy students was found to be as effective as the wet laboratory^47^. Students who used the virtual microbiology laboratory had a greater understanding of microbiology concepts, demonstrated proficiency and better performance^2^. Virtual simulation operation was a teaching tool found effective in teaching clinical skills as well and garnered positive response from the students^48^. Immersive virtual reality could enable surgical skills acquistion comparitive to the cadaver laboratory in orthopaedic residents while significantly reducing cost^49^. Even complicated skills such as mechanical ventillation and laparoscopy could be learnt using virtual reality^40,44,47,50–53^. Overall, VR laboratories not only help the students learn procedures and concepts, they also promote skills development and muscle memory. They facilitate student autonomy by removing the barriers of access, time limitation, number of attempts etc. and allow for personalised unsupervised learning whereby students with different skill levels can pace it based on their individual progress and needs^3,54^.

Student perception of VR laboratories is generally positive in our study as well as other studes and is seen as a unique and engaging learning experience^52,55^. Although most virtual laboratories have garnered positive feedback, some students have indicated that the VR platform was not easy to use. They have indicated that they need sufficient practice to be able to handle the controls in the VR laboratory comfortably^10^. Once they master the controls, they get very comfortable in using the laboratory. Some students felt uncomfortable or disoriented while using the VR technology experiencing motion sickness and visual discomfort^40,56^. Common limitations of VR laboratory are mostly associated with the use of gear (31%), time taken to get familiar (21%) and nausea (15%)^22^. With familiarity, students would be able to use the VR laboratories to their maximum potential to complement their learning. While most of the feedback on VR platforms for skills training is positive, students have still indicated that they would like to practice on real patients and real animals^48,49^. One reason for this is the lack of a realistic feeling and engagement. One way to overcome this is by creating realistic virtual environments and engaging multiple senses including haptic feedback. Using VR platforms for practice, as a preview to the face to face sessions and to reduce the laboratory or clinical time would be perceived positively rather than replacing the real experiences in the laboratories, animals or patients^57^.

Although our VR laboratory was developed for biomedical science students, it is extended to students of other health professions like medicine, pharmacy, biotechnology etc. In future, the laboratory can be further enhanced to include visualisation of enhanced microbial features, different infection scenarios and biosafety level three microbes to make it more interesting and meaningful. Future research may focus on refining the instructional design principles, integrating artificial intelligence for adaptive learning experiences, using advanced haptic technology and exploring the effectiveness of VR labs in different educational contexts.

## Conclusions

The project aimed at developing a virtual reality-based microbiology laboratory to complement our physical laboratory practical sessions. This is a pioneering project and the VR laboratory was designed in-house through a collaborative effort between different disciplines. The development of the laboratory was based on ADDIE model of instructional design starting with the needs analysis, systematic design and concluding with an evaluation of the project. The VR laboratory with head mounted display had six experiments for the students to practice their skills. The students perceived this VR laboratory to be useful and appreciated that it simulated their physical laboratory. They indicated that this helped build their knowledge and practical skills. This helped enhance the student learning experience whereby they can engage in authentic tasks, simulating the physical laboratory and protocols. This also provided flexibility to personalise student learning according to their need, time and pace in a safe environment.

Our experimental design and implementation of the VR laboratory can be adopted by other laboratory-intensive programmes. Our laboratory can serve as a model to develop similar VR-based laboratories in different subject areas. Although this laboratory was developed for one programme, this can be extended to all students in the university studying microbiology as a subject. In future, more experiments can be added to the platform and this can also be extended to other sciences as well. Virtual laboratories can also support online degree programmes and facilitate practical skills development in distant learners.

### Supplementary Information

Supplementary Information.

## Data Availability

All data generated or analysed during this study are included in this article. Additional information can be addressed to the corresponding author upon reasonable request.
