# Supplementary material for: A 3-D interactive microbiology laboratory via virtual reality for enhancing practical skills
Source: Sci Rep. 2024 Jun 4;14:12809. doi: 10.1038/s41598-024-63601-y (PMC11150509; doi:10.1038/s41598-024-63601-y)
Supplement: Supplementary file 1 — Supplementary Information. [file 41598_2024_63601_MOESM1_ESM.docx]

Overview of the VR microbiology laboratory

<https://www.youtube.com/watch?v=eKodQ5dKB04>
